# Complexity analysis of cold chain transportation in a vaccine supply chain considering activity inspection and time-delay

**DOI:** 10.1186/s13662-020-03173-z

**Published:** 2021-01-09

**Authors:** Daoming Dai, Xuanyu Wu, Fengshan Si

**Affiliations:** 1 School of Management Science and Engineering, Anhui University of Finance and Economics, Bengbu, 233030 China; 2 School of Management, Hefei University of Technology, Hefei, 230009 China

**Keywords:** Vaccine supply chain, Cold chain transportation, Time-delay, Neimark–Sacker bifurcation, Chaos

## Abstract

The development of COVID-19 vaccine is highly concerned by all countries in the world. So far, many kinds of COVID-19 vaccines have entered phase III clinical trial. However, it is difficult to deliver COVID-19 vaccines efficiently and safely to the areas affected by the epidemic. This paper focuses on vaccine transportation in a supply chain model composed of one distributor and one retailer (clinic or hospital), in which the distributor procures COVID-19 vaccines from the manufacturer and then resells them to the retailer. Distributor detects the activity level of the vaccines, and retailer is responsible for transportation of the vaccines. Firstly, we establish a difference equations model with time-delay. Secondly, we investigate the impact of time-delay on the stability of vaccine supply chain. In addition, we explore the influence of decision adjustment speed of the distributor (or retailer) on the stability of vaccine supply chain. Finally, we verify the theoretical results by a two-dimensional bifurcation diagram, the largest Lyapunov exponent, entropy, and domain of attraction. The results show that when the decision delay-time or the adjustment speed of decision variables exceeds a certain threshold, it brings a negative impact on the stability of vaccine supply chain system. The stability domain of the system shrinks as customers’ sensitivity to cold chain transportation decreases and by contrast expends as customers’ sensitivity to vaccine prices decreases. When the vaccine supply chain is in a state of chaos, the effect of external control over the system is superior to that of internal control over the system.

## Introduction

It is imperative to develop vaccines to prevent the rapidly spreading COVID-19 epidemic. Fortunately, up to now, many vaccine candidates of COVID-19 have been progressing at an unparalleled speed. 165 of them have been in the exploration or preclinical stage, 26 vaccine candidates have entered the clinical stage, and 6 of them with the fastest progress had even entered the clinical stage III. Three COVID-19 vaccines have been approved for clinical test III in China, among which Chen Wei’s team focusses on the adenovirus vector vaccine.

It is very important how the vaccine is in time delivered to the patients infected with the virus. However, the transportation of vaccines is much more complicated than ordinary commodities. Transportation of the COVID-19 vaccines from the production plant to the final retailers within a few days is a complex system including storage factories, cargo stations, airplanes, warehouses, and so on. Therefore it is very urgent to build a safe global vaccine supply chain. Once COVID-19 vaccines are available, there must be a coordinated global strategy to ensure that they are delivered to millions of infected patients as quickly and safely as possible. Although the logistics industry is hard by the COVID-19 epidemic, the air cargo industries work hard to make the delivery process smoother. TIACA (The International Air Cargo Association) and the cross-industry cooperation platform Pharma.Aero have acted in advance to jointly compile guidelines for the global air cargo industries to realize the safe transportation of the COVID-19 vaccines. Based on the status quo that “the world is working hard to build a safe and efficient supply chain to ensure that the COVID-19 vaccines reach delivery place safely”, the interesting conclusions obtained in this paper may provide some references for the transportation of the COVID-19 vaccines.

Vaccine supply chain has been studied from qualitative perspective. Evelot et al. [1] reviewed the relevant literature and found that the current research on vaccine supply chain mainly focuses on the following four aspects: vaccine quality, demand, allocation, and transport. Haidari et al. [2] found that an unmanned aerial system (UAS) to transport vaccines could improve the safety of vaccines and reduce costs. Veronica et al. [3] discovered that the inadvertent freezing of vaccine was an overlooked problem in the process of vaccine transportation. Sarley et al. [4] documented the work that the backward vaccine supply chain with Lagos State government was updated. Huang et al. [5] conducted a pilot experiment in which the health zone (HZ) was set to optimize the vaccine supply chain and assessed the incremental financial requirements for establishing a new system.

Many researchers have paid enormous attention to vaccine supply chain coordination from operation management perspective. Buyuktahtakm et al. [6] proposed a new epidemics-logistics mixed-integer programming (MIP) model to provide explicit intervention timing and intensity for these most affected countries. Abrahams et al. [7] presented a new binary integer programming model and a genetic algorithm to solve the complex scheduling problem in vaccine transportation. Arifoglu et al. [8] investigated the impact of self-interested consumers and yield uncertainty on the inefficiency of the influenza vaccine supply chain and revealed that government intervention could solve the inefficient problem of influenza vaccine supply chain. Cho [9] tried to determine the optimal composition of Influenza vaccines when vaccine production was uncertain. Dai et al. [10] investigated an influenza vaccine supply chain composed of a manufacturer and a retailer, and analyzed late deliveries and lost sales. Lin et al. [11] established a vaccine supply chain consisting of a distributor and a retailer, and discussed the impact of retailer’s inspection at the end of transportation on the original decision-making of distributor. Niu et al. [12] constructed two representative transportation channel structures, established the game theory models, and derived the vaccine price equilibrium.

Some researchers have investigated in supply chain with methods of system dynamics. Ma and Xie [13] showed complex dynamic characteristics of the evolution process in a supply chain. Li et al. [14] analyzed the influence of different parameter values on the stability and utility of low-carbon supply chain system by means of a two-dimensional bifurcation diagram, parameter plot basin, the domain of attraction, and chaos attractor. Elsadany et al. [15] focused on the price and quantity competition in a mixed duopoly game and explored the dynamical behaviors of the models. Scholars have also found similar phenomena in the vaccine supply chain. Duijzer et al. [16] used the SIR model and nonlinear dynamics of epidemic to formulate disease progression and analyzed the best time for vaccination. Duijzer et al. [17] established a differential equation of epidemic time course and analyzed the relation between the “herd effect” (when people may escape infection without being vaccinated) and vaccination fraction. Dushoff et al. [18] established an epidemic mathematical model and found that a small change of the parameter value in the vaccine supply chain would make a huge change to the optimal vaccination strategy. The existing literature mainly focuses on the analysis of complex dynamic characteristics of vaccination rate in a supply chain. However, there is little literature on complex analysis of vaccine transportation in a supply chain.

In this paper, we investigate cold transportation in a vaccine supply chain consisting of a distributor and a retailer, who are of bounded rationality. The entropy theory is used to analyze the complex dynamic characteristics of vaccine supply chain according to different parameters, such as sensitivity coefficient of vaccine retail price, sensitivity coefficient of cold chain transportation, and the adjustment speed of make decision variables. This paper is organized as follows. In Sect. 2, we make description and assumptions of the problem. In Sect. 3, we formulate the problem discussed. In Sect. 4, we obtain the conditions for the bifurcation of the system caused by time delay. In Sect. 5, we discuss in detail the simulation results. In Sect. 6, we analyze control of the chaotic system, and Sect. 7 concludes the paper.

## Description and assumptions of the problem

### Model construction and assumptions

In this paper, we mainly consider cold transportation in a vaccine supply chain consisting of a distributor and a retailer in which the for-profit distributor procures vaccines from the pharmaceutical manufacturer and then resells them to the retailer. To ensure effective vaccination for every patient infected, distributor must carry out quality sampling of vaccines to be delivered, and the retailer is responsible for transportation of the vaccines and retails them to the infected patients. Because vaccines are sensitive to temperature, retailer must choose a cold chain to transport them.

Our main assumptions are as follows:

(i) The distributor and the retailer are bounded rational.
(ii) We only consider one-time investment. If the quality sampling level for vaccines is *y*, then the distributor incurs the investment cost $\frac{k_{1}y^{2}}{2}$. If cold chain transportation level is *b*, then the retailer incurs the investment $\mathrm{cost} \frac{k_{2}b^{2}}{2}$, where $k_{1}$ and $k_{2}$ are cost coefficients [19].
(iii) To ensure that the distributor and retailer can make normal profits, let $p > w > c$.

### Symbolic description

The meanings of $\beta,p,w,A,B,y,b$, and *c* are described concisely in Table 1.

**Table 1:** The parameters description for system

Parameter	Symbolic description
*β*	Sensitivity coefficient of consumers to the retail price of vaccines.
*p*	Retail price of unit vaccine.
*w*	Wholesale price of unit vaccine.
*c*	Unit sale cost of vaccine.
*A*	Sales saturate asymptote.
*B*	Cold chain transport sensitivity.
*y*	The vaccine activity inspection level.
*b*	The cold chain transportation level.

The functional form of market demand can be written as follows:

1 $$ D = (1 - \beta p) \biggl[ A + \frac{y^{r}b^{n}}{B} \biggr]. $$

## Multiperiod decision-making game model with delay-time

The profit functions of the distributor and retailer are expressed as follows, respectively:

2 $$\begin{aligned} &\pi _{m} = (w - c) (1 - \beta p) \biggl[ A + \frac{y^{r}b^{n}}{B} \biggr] - \frac{k_{1}y^{2}}{2}, \end{aligned}$$

3 $$\begin{aligned} &\pi _{r} = (p - w) (1 - \beta p) \biggl[ A + \frac{y^{r}b^{n}}{B} \biggr] - \frac{k_{2}b^{2}}{2}. \end{aligned}$$

From Eqs. (2) and (3), the decision variable of the distributor is the vaccine activity inspection level *y*, the retailer’s decision variable is the cold chain transportation level *b*, the marginal profit function can be written as

4 $$ \textstyle\begin{cases} \frac{\partial \pi _{m}}{\partial y} = (w - c)(1 - \beta p) ( \frac{ry^{r - 1}b^{n}}{B} ) - k_{1}y, \\ \frac{\partial \pi _{r}}{\partial b} = (p - w)(1 - \beta p) ( \frac{ny^{r}b^{n - 1}}{B} ) - k_{2}b. \end{cases} $$

In the face of unfamiliar viruses, the research and development of vaccines need to invest much capital. Therefore the distributor and retailer must make an appropriate decision to reduce risk, that is, they adjust the next decision according to the current marginal profit. When the marginal profit of distributor or retailer is positive (or negative), this means that the increase of decision variables of distributor or retailer can increase (or reduce) the profit at the next period. Due to bounded rational distributor and retailer, the values of decision variables in period $t+ 1$ are the values of decision variables at period t plus the changes of decision variables at period t [20]:

5 $$ \textstyle\begin{cases} y(t + 1) = y(t) + v_{1}y(t)\frac{\partial \pi _{m}}{\partial y}, \\ b(t + 1) = b(t) + v_{2}b(t)\frac{\partial \pi _{r}}{\partial b}, \end{cases} $$

where $v_{1}$ and $v_{2}$ are the adjustment speeds of decision variables of the distributor and retailer, respectively.

Even though many scholars have investigated the vaccine supply chain, they assume that decision-makers make decisions instantaneously. However, this assumption in practice seems not realistic. The decision-makers cannot receive enough information in time when an epidemic breaks out. So we introduce the time-delay parameter into differential equations. The meaning of *τ* is the interval between the time when decision-maker should make a decision and the time when decision-maker takes a decision. We establish model I:

6 $$ \textstyle\begin{cases} y(t + 1) = y(t) + v_{1}y(t) [ (w - c)(1 - \beta p) ( \frac{ry^{r - 1}(t - \tau )b^{n}(t - \tau )}{B} ) - k_{1}y(t - \tau ) ], \\ b(t + 1) = b(t) + v_{2}b(t) [ (p - w)(1 - \beta p) ( \frac{ny^{r}(t - \tau )b^{n - 1}(t - \tau )}{B} ) - k_{2}b(t - \tau ) ]. \end{cases} $$

## Existence and local stability of Neimark–Sacker bifurcation

### Positive equilibrium points and characteristic equation of model I

By calculation we obtain two equilibrium points of model I:

$$ E_{1}(0,0),E_{2} \bigl( e^{\frac{\log ( \frac{k_{2}^{n}z_{1}^{n - 2}}{k_{1}^{n - 2}z_{2}^{n}} )}{2(n + r - 2)}},e^{\frac{\log ( \frac{k_{1}^{r}z_{2}^{r - 2}}{k_{2}^{r - 2}z_{1}^{r}} )}{2(n + r - 2)}} \bigr), $$

where

$$ \begin{aligned} &z_{1} = \frac{c\beta pr + rw - cr - \beta prw}{B}, \\ &z_{2} = \frac{np + \beta pnw - \beta p^{2}n - nw}{B}. \end{aligned} $$

It is not hard to see that only $E_{2}$ is a positive equilibrium point. The point $E_{1}$ is nonpositive, and it may be an unstable equilibrium point. We can solve the Jacobian matrix of each equilibrium point to verify the observation.

We obtain the Jacobian matrix *J* of model I and judge its stability according to the magnitude of its eigenvalues. If all the eigenvalues of the Jacobian matrix are less than 1, then this equilibrium point is stable;otherwise, it is unstable.

The Jacobian matrix *J* of model I can be written as

$$ J = \begin{vmatrix} l_{1} & 0 \\ 0 & l_{2} \end{vmatrix}, $$

where

$$ \begin{aligned} &l_{1} = 1 + v_{1} \biggl[ (w - c) ( 1 - \beta p ) \biggl( \frac{r ( y^{*} )^{r - 1} ( b^{*} )^{n}}{B} \biggr) - k_{1}y^{*} \biggr], \\ &l_{2} = 1 + v_{2} \biggl[ ( p - w ) ( 1 - \beta p ) \biggl( \frac{n ( y^{*} )^{r} ( b^{*} )^{n - 1}}{B} \biggr) - k_{2}b^{*} \biggr]. \end{aligned} $$

The Jacobian matrix $J_{1}$ at the equilibrium point $E_{1}(0,0)$ can be written as

$$ J_{1} = \begin{vmatrix} 1 & 0 \\ 0 & 1 \end{vmatrix}. $$

We can see that the eigenvalues of $J_{1}$ are not all less than 1, so the equilibrium point $E_{1}(0,0)$ is an unstable equilibrium point. Similarly, it can be proved that only the equilibrium point $E_{2}$ is a stable equilibrium point of model I.

For brevity, let $u_{1} = y(t) - y^{*},u_{2} = b(t) - b^{*}$, and we can transform the stability of the model I at the equilibrium point $E_{2}$ to the stability at the point (0,0), $y(t) = u_{1} + y^{*},b(t) = u_{2} + b^{*}$. We use the Taylor expansion to expand Eq. (6) at the equilibrium point $E_{2}$:

7 $$ \textstyle\begin{cases} y(t + 1) = t_{1}y(t) + s_{1}y(t - \tau ) + s_{2}b(t - \tau ), \\ b(t + 1) = t_{2}b(t) + s_{3}y(t - \tau ) + s_{4}b(t - \tau ), \end{cases} $$

where

$$\begin{aligned} &t_{1} = 1 + v_{1} \biggl[ (w - c) ( 1 - \beta p ) \biggl( \frac{r ( y^{*} )^{r - 1} ( b^{*} )^{n}}{B} \biggr) \biggr], \\ &s_{1} = v_{1}y^{*} \biggl[ (w - c) ( 1 - \beta p ) \biggl( \frac{r(r - 1) ( y^{*} )^{r - 2} ( b^{*} )^{n}}{B} \biggr) - k_{1} \biggr], \\ &s_{2} = v_{1}y^{*} \biggl[ (w - c) ( 1 - \beta p ) \biggl( \frac{rn ( y^{*} )^{r - 1} ( b^{*} )^{n - 1}}{B} \biggr) \biggr], \\ &t_{2} = 1 + v_{2} \biggl[ ( p - w ) ( 1 - \beta p ) \biggl( \frac{n ( y^{*} )^{r} ( b^{*} )^{n - 1}}{B} \biggr) \biggr], \\ &s_{3} = v_{2}b^{*} \biggl[ ( p - w ) ( 1 - \beta p ) \biggl( \frac{nr ( y^{*} )^{r - 1} ( b^{*} )^{n - 1}}{B} \biggr) \biggr], \\ &s_{4} = v_{2}b^{*} \biggl[ ( p - w ) ( 1 - \beta p ) \biggl( \frac{n(n - 1) ( y^{*} )^{r} ( b^{*} )^{n - 2}}{B} \biggr) - k_{2} \biggr]. \end{aligned}$$

Next, the characteristic determinant of model I can be written as

$$ \begin{vmatrix} \lambda - t_{1} - s_{1}e^{ - \lambda \tau } & - s_{2}e^{ - \lambda \tau } \\ - s_{3}e^{ - \lambda \tau } & \lambda - t_{2} - s_{4}e^{ - \lambda \tau } \end{vmatrix}. $$

Then we obtain the characteristic equation of model I

8 $$ \lambda ^{2} - ( t_{1} + t_{2} )\lambda + t_{1}t_{2} + \bigl[ ( - s_{4} - s_{1} )\lambda + t_{1}s_{4} \bigr]e^{ - \lambda \tau } + ( s_{1}t_{2} + s_{1}s_{4} )e^{ - 2\lambda \tau } = 0. $$

### Conditions for local stability at equilibrium point $E_{2}$ when $\tau =0$

When $\tau = 0$, Eq. (8) can be simplified as follows:

9 $$ \lambda ^{2} - ( t_{1} + t_{2} + s_{4} + s_{1} )\lambda + t_{1}t_{2} + + t_{1}s_{4} + s_{1}t_{2} + s_{1}s_{4} = 0. $$

According to the Routh–Hurwitz criterion, if $(H_{2}): - ( t_{1} + t_{2} + s_{4} + s_{1} ) > 0$, $t_{1}t_{2} + + t_{1}s_{4} + s_{1}t_{2} + s_{1}s_{4} > 0$, and $- ( t_{1} + t_{2} + s_{4} + s_{1} ) > t_{1}t_{2} + t_{1}s_{4} + s_{1}t_{2} + s_{1}s_{4}$, then the equilibrium point $E_{2}$ is locally asymptotically stable.

### Conditions for local stability at equilibrium point $E_{2}$ when $\tau >0$

Multiplying both sides of Eq. (8) by $e^{\lambda \tau } $, we obtain

10 $$ ( - s_{4} - s_{1} )\lambda + t_{1}s_{4} + \bigl[ \lambda ^{2} - ( t_{1} + t_{2} )\lambda + t_{1}t_{2} \bigr]e^{\lambda \tau } + ( s_{1}t_{2} + s_{1}s_{4} )e^{ - \lambda \tau } = 0. $$

Let $\lambda = i\omega (\omega > 0)$ be a root of Eq. (10). Then

11 $$ \textstyle\begin{cases} \Delta _{1}\cos (\omega \tau ) + \Delta _{2}\sin (\omega \tau ) = \Delta _{4}, \\ - \Delta _{2}\cos (\omega \tau ) + \Delta _{3}\sin (\omega \tau ) = \Delta _{5}, \end{cases} $$

where

$$\begin{aligned} &\Delta _{1} = - \omega ^{2} + t_{1}t_{2} + s_{1}t_{2} + s_{1}s_{4}, \\ &\Delta _{2} = \omega ( t_{1} + t_{2} ), \\ &\Delta _{3} = - \omega ^{2} + t_{1}t_{2} - s_{1}t_{2} - s_{1}s_{4}, \\ &\Delta _{4} = - s_{1}t_{2} - s_{1}s_{4} - t_{1}s_{4}, \\ &\Delta _{5} = s_{4} + s_{1}. \end{aligned}$$

By Eq. (11) we obtain

12 $$ \textstyle\begin{cases} \cos (\omega \tau ) = \frac{ - \omega ^{2}\Delta _{4} - \omega ( t_{1} + t_{2} )\Delta _{5} + q_{2}\Delta _{4}}{\omega ^{4} + \omega ^{2} [ ( t_{1} + t_{2} )^{2} - ( q_{1} + q_{2} ) ] + q_{1}q_{2}}, \\ \sin (\omega \tau ) = \frac{ - \omega ^{2}\Delta _{5} + \omega ( t_{1} + t_{2} )\Delta _{4} + q_{1}\Delta _{5}}{\omega ^{4} + \omega ^{2} [ ( t_{1} + t_{2} )^{2} - ( q_{1} + q_{2} ) ] + q_{1}q_{2}}, \end{cases} $$

where

$$\begin{aligned} &q_{1} = t_{1}t_{2} + s_{1}t_{2} + s_{1}s_{4}, \\ &q_{2} = t_{1}t_{2} - s_{1}t_{2} - s_{1}s_{4}. \end{aligned}$$

By Eq. (12) we have

13 $$ \omega ^{8} + \omega ^{6}m_{1} + \omega ^{4}m_{2} + \omega ^{2}m_{3} + \omega m_{4} + m_{5} = 0, $$

where

$$\begin{aligned} &m_{1} = 2 ( t_{1} + t_{2} )^{2} - 2 ( q_{1} + q_{2} ), \\ &m_{2} = \bigl[ ( t_{1} + t_{2} )^{2} - ( q_{1} + q_{2} ) \bigr]^{2} + 2q_{1}q_{2} - \Delta _{4}^{2} - \Delta _{5}^{2}, \\ &m_{3} = \bigl[ 2q_{1}q_{2} ( t_{1} + t_{2} )^{2} - ( q_{1} + q_{2} ) \bigr] - ( t_{1} + t_{2} )^{2} \bigl( \Delta _{5}^{2} + \Delta _{4}^{2} \bigr) + 2 \Delta _{4}^{2}q_{2} + 2\Delta _{5}^{2}q_{1}, \\ &m_{4} = 2 ( t_{1} + t_{2} )\Delta _{4}\Delta _{5} ( q_{2} - q_{1} ), \\ &m_{5} = ( q_{1}q_{2} )^{2} - q_{2}^{2}\Delta _{4}^{2} - q_{1}^{2}\Delta _{5}^{2}. \end{aligned}$$

Define $f(\omega ) = \omega ^{8} + \omega ^{6}m_{1} + \omega ^{4}m_{2} + \omega ^{2}m_{3} + \omega m_{4} + m_{5} = 0$. To derive the main results of this paper, we assume that

$(H_{2}):f(\omega )$ has *k* positive roots, denoted by $f_{1},f_{2},\ldots,f_{k},0 < k \le 8$. From Eq. (12) we have

14 $$ \begin{aligned} &\tau _{i}^{(j)} = \frac{1}{\omega _{i}}\arccos \biggl\{ \frac{ - \omega ^{2}\Delta _{4} - \omega ( t_{1} + t_{2} )\Delta _{5} + q_{2}\Delta _{4}}{\omega ^{4} + \omega ^{2} [ ( t_{1} + t_{2} )^{2} - ( q_{1} + q_{2} ) ] + q_{1}q_{2}} \biggr\} + \frac{2j\pi }{\omega _{k}},\\ &\quad i = 1,2,3, \ldots,k;j = 1,2, \ldots. \end{aligned} $$

Let

15 $$ \tau _{0} = \min \bigl\{ \tau _{i}^{(j)}, i = 1,2, \ldots,k;j = 0,1 \cdots \bigr\} = \min \bigl\{ \tau _{i}^{(0)}, i = 1,2, \ldots,k \bigr\} = \tau _{i_{0}}^{(0)}. $$

Differentiating both sides of Eq. (10) with respect to *τ*, we get

16 $$ \biggl[ \frac{d\lambda }{d\tau } \biggr]^{ - 1} = \frac{ ( - s_{4} - s_{1} ) + 2\lambda e^{\lambda \tau }}{ ( t_{1} + t_{2} )e^{\lambda \tau } + \lambda e^{ - \lambda \tau } ( s_{1}t_{2} + s_{1}s_{4} ) - \lambda e^{\lambda \tau } [ \lambda ^{2} - ( t_{1} + t_{2} )\lambda + t_{1}t_{2} ]} - \frac{\tau }{\lambda }. $$

When $\tau = \tau _{0}$, substituting $\lambda = i\omega _{0}$ into Eq. (16), we get

17 $$ \operatorname{Re} \biggl[ \frac{d\lambda }{d\tau } \biggr]_{\tau = \tau _{0}}^{ - 1} = \frac{R_{1}R_{2} + S_{1}S_{2}}{R_{1}^{2} + S_{1}^{2}}, $$

where

$$\begin{aligned} &R_{1} = ( t_{1} + t_{2} ) \bigl( 1 - \omega _{0}^{2} \bigr)\cos ( \omega _{0}\tau _{0} ) + \bigl[ \omega _{0} ( s_{1}t_{2} + s_{1}s_{4} ) - \omega _{0}^{3} + \omega _{0}t_{1}t_{2} \bigr]\sin ( \omega _{0}\tau _{0} ), \\ &S_{1} = \bigl[ \omega _{0} ( s_{1}t_{2} + s_{1}s_{4} ) - \omega _{0}^{3} - \omega _{0}t_{1}t_{2} \bigr]\cos ( \omega _{0}\tau _{0} ) + ( t_{1} + t_{2} ) \bigl( 1 - \omega _{0}^{2} \bigr)\sin ( \omega _{0}\tau _{0} ), \\ &R_{2} = ( - s_{4} - s_{1} ) - 2\omega _{0}\sin ( \omega _{0}\tau _{0} ), \\ &S_{2} = 2\omega _{0}\cos ( \omega _{0}\tau _{0} ). \end{aligned}$$

To ensure the condition of the occurrence for Neimark–Sacker bifurcation, we introduce have the following hypothesis:

$$ ( H_{3} ):\quad R_{1}R_{2} + S_{1}S_{2} \ne 0. $$

Then we have the following results.

#### Theorem 1

*For Model I*, *if conditions* $(H_{1}), (H_{2})$, *and* ($H_{3}$) *hold*, *then the equilibrium point* $E_{2}$ *is asymptotically stable for* $\tau \in [0,\tau _{0})$; *when* $\tau = \tau _{0}$, *model I undergoes a Neimark–Sacker bifurcation at equilibrium point* $E_{2}$, *and it is unstable at equilibrium point* $E_{2}$ *when* $\tau > \tau _{0}$.

#### Proof

The proof of Theorem 1 is completed by investigating the distribution of the roots of Eq. (8). To analyze the distribution of roots of the transcendental Eq. (8), we need the following lemmas. □

#### Lemma 1

*For the transcendental equation*

18 $$\begin{aligned} \begin{aligned} P \bigl( \lambda,e^{ - \lambda \tau _{1}}, \ldots,e^{ - \lambda \tau _{m}} \bigr) ={}& \lambda ^{n} + p_{1}^{(0)}\lambda ^{n - 1} + \cdots + p_{n - 1}^{(0)}\lambda + p_{n}^{(0)} \\ & {}+ \bigl[ p_{1}^{(1)}\lambda ^{n - 1} + \cdots + p_{n - 1}^{(1)}\lambda + p_{n}^{(1)} \bigr]e^{ - \lambda \tau _{1}} + \cdots \\ & {}+ \bigl[ p_{1}^{(m)}\lambda ^{n - 1} + \cdots + p_{n - 1}^{(m)}\lambda + p_{n}^{(m)} \bigr]e^{ - \lambda \tau _{m}}, \end{aligned} \end{aligned}$$

*as* $( \tau _{1},\tau _{2},\tau _{3}, \ldots,\tau _{m} )$ *vary*, *the sum of orders of the zeros of* $P ( \lambda,e^{ - \lambda \tau _{1}}, \ldots,e^{ - \lambda \tau _{m}} )$ *in the open right half*-*plane can change*, *and only a zero appears on or crosses the imaginary axis* [21].

When $\tau = 0$, Eq. (8) can be rewritten as

19 $$ \lambda ^{2} - ( t_{1} + t_{2} + s_{4} + s_{1} )\lambda + t_{1}t_{2} + + t_{1}s_{4} + s_{1}t_{2} + s_{1}s_{4} = 0. $$

From the Routh–Hurwitz criterion we know that a necessary and sufficient condition for all roots of Eq. (19) to have negative real parts is

$$\begin{aligned} &(H_{1}):\quad {-} ( t_{1} + t_{2} + s_{4} + s_{1} ) > 0,\qquad t_{1}t_{2} + + t_{1}s_{4} + s_{1}t_{2} + s_{1}s_{4} > 0,\\ &\phantom{(H_{1}):\quad} {-} ( t_{1} + t_{2} + s_{4} + s_{1} ) > t_{1}t_{2} + t_{1}s_{4} + s_{1}t_{2} + s_{1}s_{4}. \end{aligned}$$

When $\tau > 0$, Eq. (8) becomes

20 $$ ( - s_{4} - s_{1} )\lambda + t_{1}s_{4} + \bigl[ \lambda ^{2} - ( t_{1} + t_{2} )\lambda + t_{1}t_{2} \bigr]e^{\lambda \tau } + ( s_{1}t_{2} + s_{1}s_{4} )e^{ - \lambda \tau } = 0. $$

*iω* is a root of Eq. (20) if and only if

21 $$ \begin{aligned} &( - s_{4} - s_{1} )i\omega + t_{1}s_{4} + \bigl[ - \omega ^{2} - ( t_{1} + t_{2} )i\omega + t_{1}t_{2} \bigr]\bigl(\cos (\omega \tau ) + i\sin (\omega \tau )\bigr) \\ &\quad + ( s_{1}t_{2} + s_{1}s_{4} ) \bigl(\cos (\omega \tau ) - i\sin (\omega \tau )\bigr) = 0. \end{aligned} $$

From Eq. (21) we obtain

22 $$ \textstyle\begin{cases} \Delta _{1}\cos (\omega \tau ) + \Delta _{2}\sin (\omega \tau ) = \Delta _{4}, \\ - \Delta _{2}\cos (\omega \tau ) + \Delta _{3}\sin (\omega \tau ) = \Delta _{5}, \end{cases} $$

which leads to

23 $$ \omega ^{8} + \omega ^{6}m_{1} + \omega ^{4}m_{2} + \omega ^{2}m_{3} + \omega m_{4} + m_{5} = 0. $$

Without loss of generality, we assume that

$( H_{2} )$: Eq. (23) has *k* positive roots, denoted by $f_{1},f_{2},\ldots,f_{k},0 < k \le 8$.

By Eq. (22) we have

24 $$ \cos (\omega \tau ) = \frac{ - \omega ^{2}\Delta _{4} - \omega ( t_{1} + t_{2} )\Delta _{5} + q_{2}\Delta _{4}}{\omega ^{4} + \omega ^{2} [ ( t_{1} + t_{2} )^{2} - ( q_{1} + q_{2} ) ] + q_{1}q_{2}}. $$

Denote

25 $$\begin{aligned} &\tau _{i}^{(j)} = \frac{1}{\omega _{i}}\arccos \biggl\{ \frac{ - \omega ^{2}\Delta _{4} - \omega ( t_{1} + t_{2} )\Delta _{5} + q_{2}\Delta _{4}}{\omega ^{4} + \omega ^{2} [ ( t_{1} + t_{2} )^{2} - ( q_{1} + q_{2} ) ] + q_{1}q_{2}} \biggr\} + \frac{2j\pi }{\omega _{i}},\\ &\quad i = 1,2, \ldots,k;j = 0.1 \cdots, \end{aligned} $$

that is, $\pm i\omega _{i}$ is a pair of purely imaginary roots of Eq. (8).

Denote

$$ \tau _{0} = \min \bigl\{ \tau _{i}^{(0)}, k = 1,2i, \ldots,k \bigr\} = \tau _{i_{0}}^{(0)}. $$

#### Lemma 2

*If conditions* ($H_{1}$) *and* ($H_{2}$) *hold*, *then for* $\tau \in [0,\tau _{0})$, *all roots of Eq*. (8) *have negative real parts*, *and for* $\tau = \tau _{i}^{(j)}\ (i = 1,2, \ldots,k;j = 0,1, \ldots )$, *Eq*. (8) *has a pair of pure imaginary roots*.

We define $\lambda (\tau ) = \alpha (\tau ) + i\omega (\tau )$ as the root of Eq. (8) satisfying $\alpha ( \tau _{i} ) = 0$ and $\omega ( \tau _{i} ) = \omega _{0}$. Multiplying both sides of Eq. (8) by $e^{\lambda \tau } $ and differentiating both sides of Eq. (8) with respect to *τ*, we get

26 $$ \biggl[ \frac{d\lambda }{d\tau } \biggr]^{ - 1} = \frac{ ( - s_{4} - s_{1} ) + 2\lambda e^{\lambda \tau }}{ ( t_{1} + t_{2} )e^{\lambda \tau } + \lambda e^{ - \lambda \tau } ( s_{1}t_{2} + s_{1}s_{4} ) - \lambda e^{\lambda \tau } [ \lambda ^{2} - ( t_{1} + t_{2} )\lambda + t_{1}t_{2} ]} - \frac{\tau }{\lambda }. $$

Then

27 $$ \operatorname{Re} \biggl[ \frac{d\lambda }{d\tau } \biggr]_{\tau = \tau _{0}}^{ - 1} = \frac{R_{1}R_{2} + S_{1}S_{2}}{R_{1}^{2} + S_{1}^{2}}. $$

Assuming that

$$ ( H_{3} ):\quad R_{1}R_{2} + S_{1}S_{2} \ne 0 \quad\text{and}\quad \operatorname{sign} \bigl(\operatorname{Re} (a + ib)\bigr) = \operatorname{sign} \bigl( \operatorname{Re} (a + ib)^{ - 1} \bigr), $$

we obtain

28 $$ \alpha ^{\prime } ( \tau _{i} ) > 0. $$

From Lemmas 1–2 we have the following results on the distribution of the roots of Eq. (8): if conditions $(H_{1}),(H_{2})$, and ($H_{3}$) hold, then for $\tau \in [ 0,\tau _{0} )$, all roots of Eq. (8) have negative real parts; and for $\tau = \tau _{0}$, Eq. (8) has a pair of pure imaginary roots; Eq. (8) has at least one pair of roots with positive real parts for $\tau > \tau _{0}$. Then combining the results obtained by Hale [22], we get Theorem 1, and the proof is complete.

## Numerical simulation

In this section, we mainly verify the theoretical results. Set $p = 4.8,w = 3.4, \beta = 0.26, c = 2,A = 3,B = 0.65,y = 2,b = 3,k_{1} = k_{2} = 0.5$. We use the largest Lyapunov exponent and entropy to measure the features of system changes. The principle of the largest Lyapunov exponent is that the system is stable when the exponent is less than zero; otherwise, it is unstable. The rules of entropy to judge the system stability are as follows: the system is in a stable state when the entropy is zero; otherwise, it is unstable.

### Neimark–Sacker bifurcation diagram caused by time-delay

**Figure 1:**
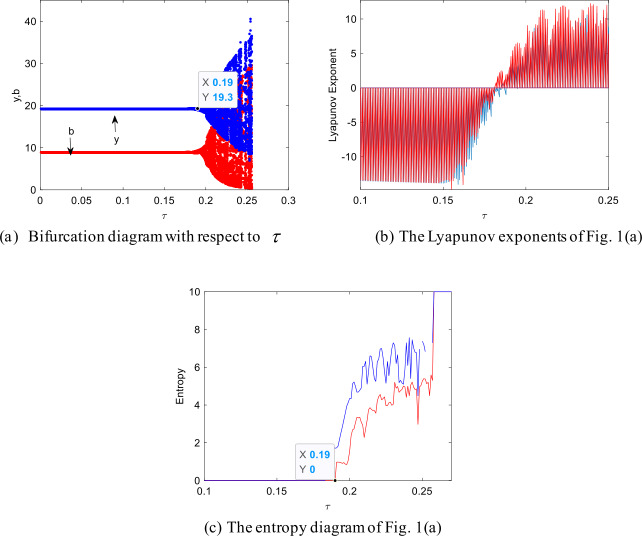
The impact of *τ* on the stability

Figures 1(a)–(c) show how the system stability changes with time-delay. As can be seen from Fig. 1(a), with the increase of *τ*, at first, Model I remains stable, then it gradually produces a Neimark–Sacker bifurcation, and, finally, it becomes chaotic. The critical point $\tau _{0}$ of bifurcation is equal to 0.19. That is, when $\tau < 0.19$, the equilibrium point $E_{2} ( y^{*},b^{*} )$ is asymptotically stable; when *τ* increases and passes through $\tau _{0}$, the model I bifurcates and loses its stability.

From Fig. 1(a) we can see that the vaccine transportation equilibrium point is locally asymptotically stable if the time-delay parameters is less than the critical value. When vaccine transportation is stable, the distributors and retailers can calmly respond to the epidemic and take reasonable decisions to ensure that the vaccine can reach its destination safely. However, once the time-delay parameter exceeds a certain threshold, the system undergoes a Neimark–Sacker bifurcation and goes into chaos. In this case, the distributor and retailer must bear the losses caused by the failure to respond to the epidemic in time. Figures 1(b) and (c) show the corresponding largest Lyapunov exponent and entropy of Fig. 1(a). When $\tau < 0.19$, the largest Lyapunov exponent is less than zero, and the entropy is equal to zero, and the system keeps a stable state; otherwise, the largest Lyapunov exponent is greater than zero, the entropy of the system continues to increase, and the system falls into chaos state.

### Bifurcation diagram caused by adjusting speed of decision variable

**Figure 2:**
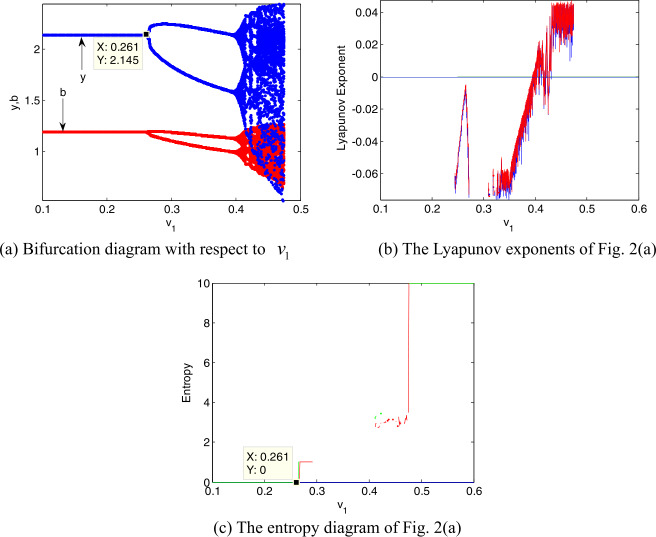
Dynamic evolution of decision variables with $v_{1}$

To further understand the dynamic characteristics of the system, in this section, we use the bifurcation diagram, largest Lyapunov exponent, and entropy to analyze the influence of the adjustment speed of decision variables on the system stability. Ceteris paribus, letting $\tau = 0.1$, the influence of time-delay on the stability of the system can be eliminated. Assuming that $v_{2} = 0.015$ and $v_{1}$ changes from 0 to 0.5, we analyze the effect of $v_{1}$ on the system stability. From Fig. 2(a) we see that when $v_{1}$ increases from 0 to 0.261, the vaccine activity inspection level *y* (indicated by yellow line) and the cold chain transportation level *b* (indicated by brown line) are asymptotically stable; when $v_{1}$ reaches 0.261, *y* and *b* start to bifurcate, and the system turns into stable cycles of period 2; as the $v_{1}$ continues to increase, the system eventually enters into a chaotic state.

**Figure 3:**
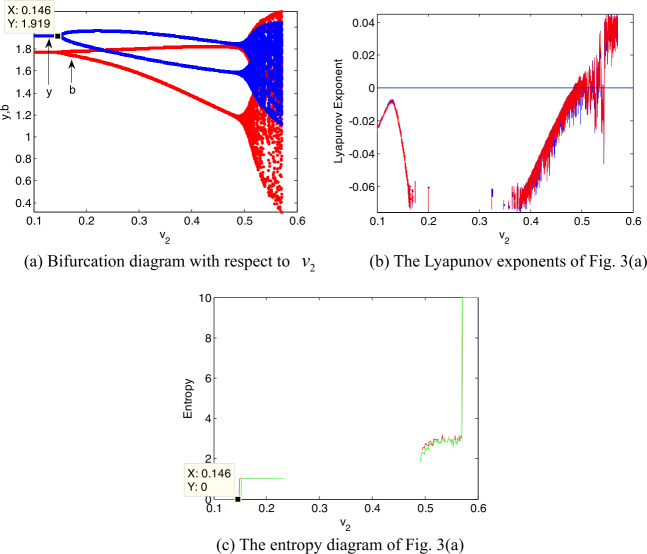
Dynamic evolution of decision variables with $v_{2}$

Figure 3(a) presents the effect of $v_{2}$ on the system stability, which is similar to Fig. 2(a). When $v_{2} \in [0,0.146)$, the system is in a stable state; when $v_{2}$ increases to 0.146, the first bifurcation occurs, and the system turns into stable cycles of period 2; with the increase of $v_{2}$, the system finally enters into chaotic state.

The largest Lyapunov exponent can further verify the dynamic characteristics of the system. Take Fig. 2(b) for an example: when the Lyapunov exponent first returns to the zero axis, the system appears the first bifurcation; when Lyapunov exponent is always greater than zero, then the system enters into a chaotic state, which is consistent with the dynamic characteristics shown in Fig. 2(a). Similarly, we also can illustrate the dynamic characteristics of the system through the change of entropy. From Fig. 2(c) we see that when $v_{1} \in (0,0.261)$, the entropy of the model I is equal to zero, and at this time, *y* and *b* are asymptotically stable; when $v_{1} > 0.261$,the entropy of model I is greater than zero, and the model I undergoes a period doubling bifurcation state; with the further increase of $v_{1}$, the entropy also continues to increase. At this time, *y* and *b* are unstable and may take multiple possible values. Due to increase of the entropy, the distributor and retailer need more additional information to make reasonable decisions. Similar insights can be obtained in Figs. 3(b) and (c).

From Figs. 2–3 we see that the faster the adjustment speed of the vaccine activity inspection level (or the cold chain transportation level), the more chaotic the vaccine supply chain. From the perspective of entropy theory, when the vaccine supply chain falls into chaos, its entropy will be high. So the distributor and the retailer may obtain additional information to choose an appropriate adjustment speed. Therefore the distributor and retailer should collect abundant market information in advance and make rational decisions to prevent the vaccine supply chain from getting into chaos. Comparing Fig. 2(a) with Fig. 3(a), the critical value of system bifurcation caused by the adjustment speed $v_{2}$ of cold chain transportation level is less than that caused by the adjustment speed $v_{1}$ of activity inspection level. This interesting phenomenon means that the reasonable adjustment range of activity inspection level is larger than that of cold chain transportation level, and consumers may be more sensitive to cold chain transportation level. Thus the improvement of the cold chain transportation level in the vaccine supply chain seems to be in the first place.

### Global stability analysis

**Figure 4:**
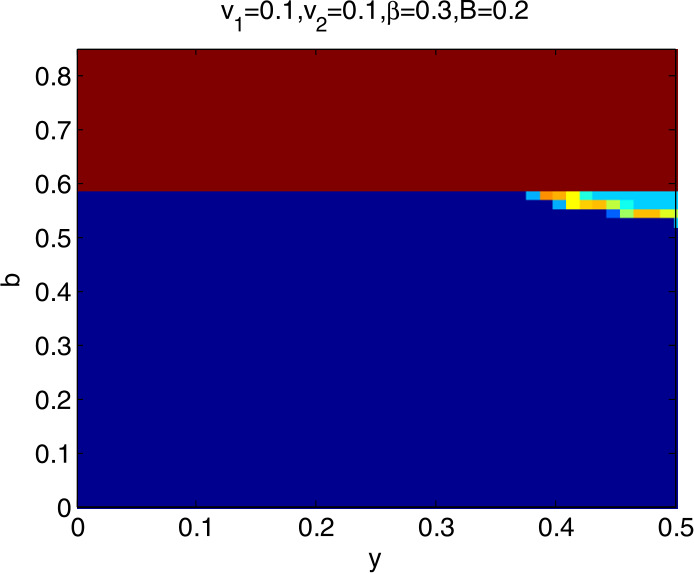
Domain of attraction of the model I

**Figure 5:**
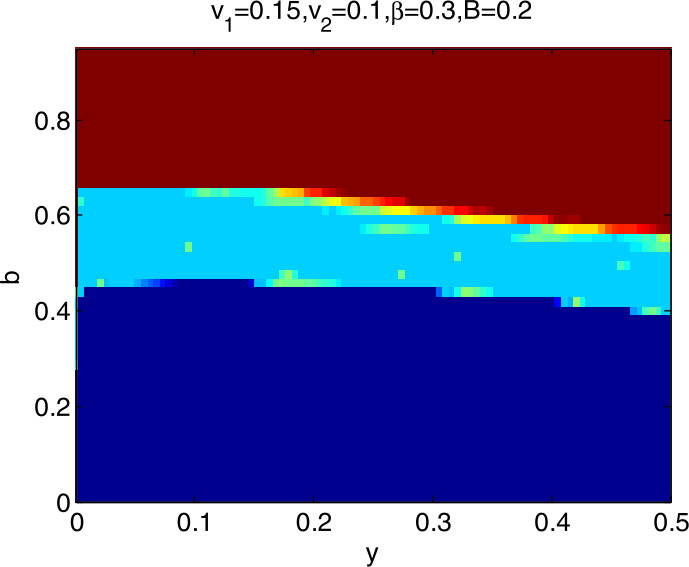
Domain of attraction of the model I when $v_{1}$ increases to 0.15

**Figure 6:**
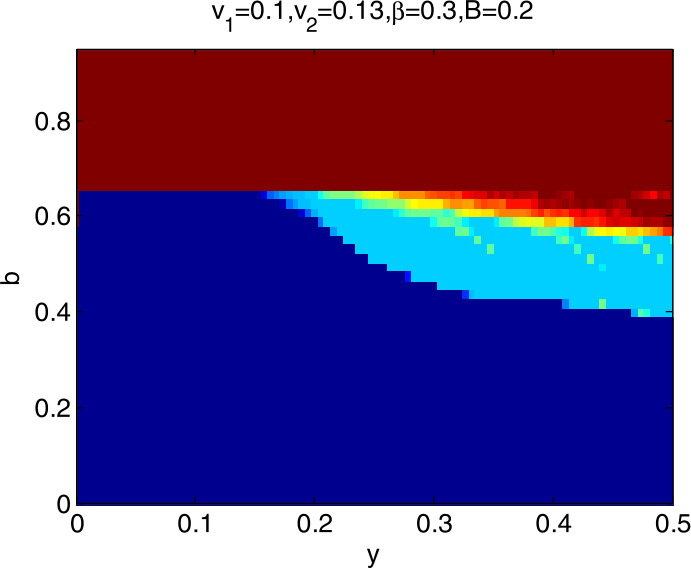
Domain of attraction of the model I when $v_{2}$ increases to 0.13

**Figure 7:**
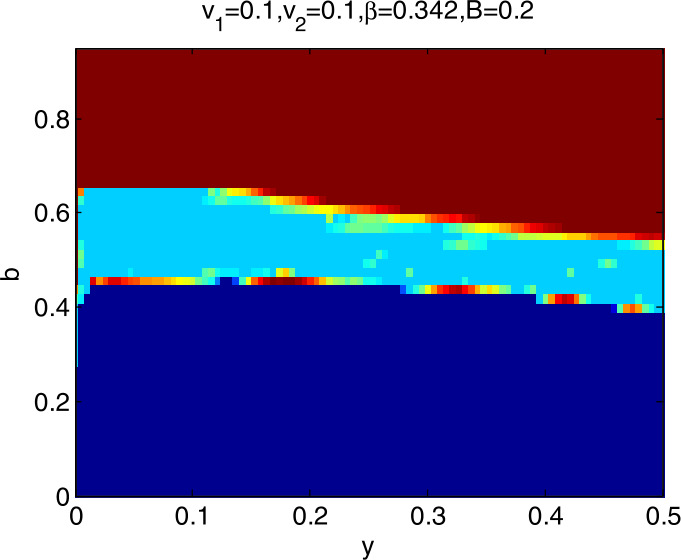
Domain of attraction of the model I when *β* increases to 0.342

**Figure 8:**
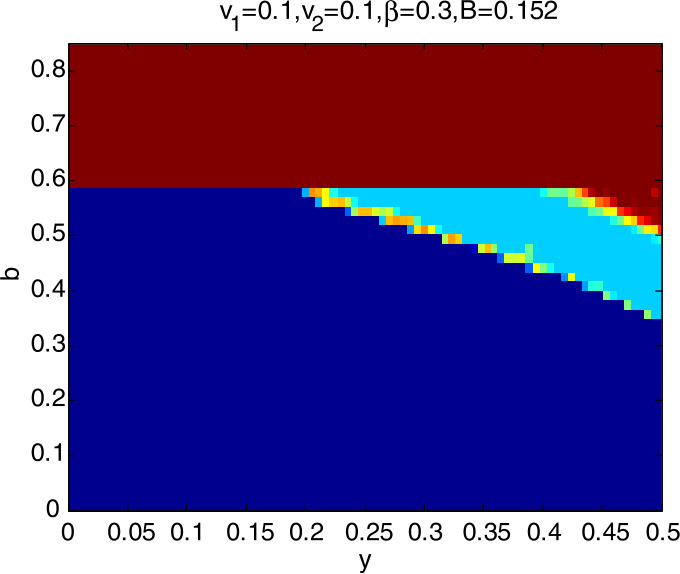
Domain of attraction of the model I when *B* increases to 0.152

To further explore the dynamic characteristics of model I with the change of $v_{1}$, $v_{2}$, *β*, and *B*, ceteris paribus, letting *y* and *b*∈ (0,1), we obtain that the domain of attraction of the model I is as in Fig. 4, in which the dark blue region denotes the stable attraction domain, light blue denotes the period-2 attraction region, and the deep red denotes the escape area. Figs. *i* ($i= 5,6,7,8$) show the domain of attraction when $v_{1}$, $v_{2}$, *β*, *B* change in turn. Comparing Fig. 4 with Figs. 5 and 6, we can see that the stable attraction domains decrease with the increase of $v_{1}$ or $v_{2}$, which is consistent with the conclusions reflected in Figs. 2 and 3. Comparing Fig. 4 with Fig. 7, the stable attraction domain decreases with the increase of *β*, which means that the lower the sensitivity of consumers to the vaccine price, the stabler the vaccine supply chain. Similarly, comparing Fig. 4 with Fig. 8, the stable attraction domain decreases with the decrease of *B*, which means that the higher the sensitivity of consumers to the cold chain transportation, the stabler the vaccine supply chain.

From Figs. 4–8 we see that the increase of consumers’ sensitivity to vaccine prices has a negative impact on vaccine transportation. It is very necessary for the government to take measures to eliminate consumers’ concerns about vaccine prices, such as providing subsidies to consumers. On the other hand, consumers’ sensitivity to cold chain transportation is beneficial to the vaccine transportation system.

## Chaos control

The vaccine transportation cannot be carried out smoothly by chaos in the vaccine supply chain, and the vaccines may be inactivated. Chaos in vaccine supply chain can be controlled by the adjustment parameter control method and variable feedback control method. The chaotic control effect of adjusting the parameters on model I is analyzed by numerical simulation. As mentioned before, ceteris paribus, when $\tau = 0.4,v_{1} = 0.35,v_{2} = 0.35$, the vaccine supply chain is in chaos.

### Adjustment parameter control method

The original vaccine supply chain system is

29 $$ \textstyle\begin{cases} y(t + 1) = y(t) + v_{1}y(t)\frac{\partial \pi _{m}}{\partial y}, \\ b(t + 1) = b(t) + v_{2}b(t)\frac{\partial \pi _{r}}{\partial b}. \end{cases} $$

The vaccine supply chain system after parameter adjustment control is as follows [23]:

30 $$ \textstyle\begin{cases} y(t + 1) = (1 - u) [ y(t) + v_{1}y(t)\frac{\partial \pi _{m}}{\partial y} ] + uy(t), \\ b(t + 1) = (1 - u) [ b(t) + v_{2}b(t)\frac{\partial \pi _{r}}{\partial b} ] + ub(t). \end{cases} $$

**Figure 9:**
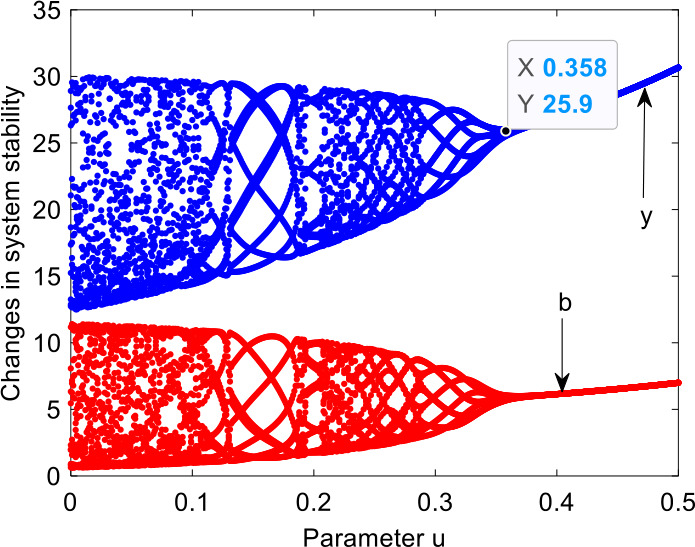
Systematic bifurcations with variation of the adjustment parameter *u*

With the variation of the adjustment parameter *u*, the system changes as shown in Fig. 9. When *u* is less than the threshold (0.358), the system is in chaotic state, which indicates that the distributor and retailer did not take any effective joint measures to control chaos. With the increase of *u*, the system reaches a stable state, which indicates that the distributor and retailer can effectively reduce chaos by taking joint measures, such as signing contracts and so on.

### Variable feedback control method

The main principle of variable feedback control method is using an equation variable as control signal to eliminate chaos. Compared with other control methods, this method has the advantages of simple controller design and strong timeliness. Therefore it is widely used in general discrete dynamic systems. Based on this, the dynamic system with new controllers is established as follows [24]:

31 $$ \textstyle\begin{cases} y(t + 1) = y(t) + v_{1}y(t)\frac{\partial \pi _{m}}{\partial y} - uy(t), \\ b(t + 1) = b(t) + v_{2}b(t)\frac{\partial \pi _{r}}{\partial b} - ub(t). \end{cases} $$

**Figure 10:**
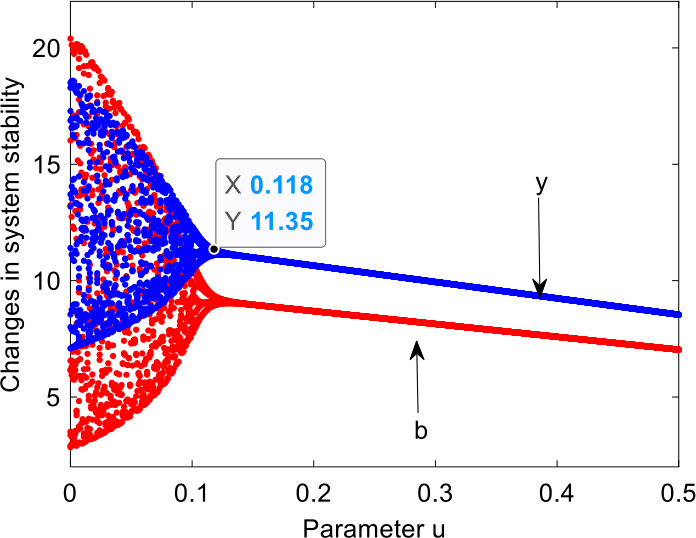
Systematic bifurcations with variation of adjustment parameter *u*

Figure 10 shows that the chaotic system gradually returns to the stable state through variable feedback control method. When *u* is less than the threshold (0.118), the system is in chaotic state, which indicates that the government does not take effective control measures for the chaotic system at this time. When $u > 0.118$, the system remains asymptotically stable, which indicates that the government takes external intervention measures to accelerate the system to the stable state and ensures stable economic development.

Comparing Fig. 9 with Fig. 10, it is obvious that the control system in Fig. 10 enters the stable state earlier than that in Fig. 9, which means that the control effect of the variable feedback control method is better than that of the adjustment parameter control method. Because of the extra cost of control, it is difficult for the distributor and retailer to actively control the chaotic vaccine supply chain. In addition, the safety of vaccines cannot be guaranteed in the chaotic vaccine supply chain, so external forces (for example, the regulation and policy of the government) usually are used to control the chaos of vaccine supply chain in time.

## Conclusion

In this study, we mainly investigated the vaccine supply chain composed of a distributor and retailer. The distributor is responsible for the vaccine activity inspection of the vaccines before they are delivered to the retailer, whereas the retailer carries out the cold chain transportation of the vaccines. In addition, the decision-making of the distributor and retailer is not instantaneous, but rather time-delayed. We analyzed the complex dynamic characteristics of the system are by using the Neimark–Sacker bifurcation diagram, attraction domain, and entropy. We used the adjustment parameter control and variable feedback control methods to control the chaotic system. We obtained the following conclusions.

(1) When the time-delay parameter $\tau \in [ 0,\tau _{0} )$, the vaccine transportation equilibrium is locally asymptotically stable. At this time, the distributor and retailer can cooperate well to ensure the vaccine activity. When the time-delay parameter $\tau \ge \tau _{0}$, the vaccines transportation system produces a Neimark–Sacker bifurcation and loses stability. (2) If the distributor and retailer adjust the decision variables too quickly, then the vaccine supply chain will bifurcate and even fall into chaos, which means that the distributor and retailer should choose appropriate adjustment speeds of the decision variables to prevent the vaccine supply chain from get into chaos. (3) The stability domain of the system shrinks as customers’ sensitivity to cold chain transportation decreases, and by contrast it expends as customers’ sensitivity to vaccine prices decreases. (4) Compared with the internal joint control of the distributor and retailer, the effect of external control, such as government intervention, will have a better control effect on the chaos of the system.

## Data Availability

The data used to support the findings of this study are available from the corresponding author upon request.
